# Assessment of Speech Perception Abilities in Cochlear Implant Children

**DOI:** 10.22038/IJORL.2022.60164.3070

**Published:** 2022-05

**Authors:** Salwa Mourad Abdelmawgoud Elsayed

**Affiliations:** *Audio-vestibular Unit, Department of Otolaryngology, Sohag Faculty of Medicine, Sohag University, Sohag, Egypt.*

**Keywords:** Cochlear implant, Cortical evoked potential, Speech perception

## Abstract

**Introduction:**

***The ability to perceive speech is a key sign of language development and normal speech. The current study was designed to measure the speech perception abilities in children with cochlear implant both in subjective and objective manners***

**Materials and Methods:**

The research has been reviewed and approved by Medical ethical Committee and Thai Clinical Trials registry Committee. Sixty children age range from five to eight years with a pre-lingual bilateral profound sensori-neural hearing loss, fitted with a cochlear implant for two years or more were included. They were divided into two equal groups {thirty children in each group}; group I with good progress in auditory training and language acquisition and group II with poor progress in auditory training and language acquisition. Speech perception abilities were evaluated subjectively via Speech perception tests and objectively by measuring cortical evoked potentials. The results of speech perception tests and cortical evoked potential were analyzed and correlated.

**Results:**

***There was a statically significant difference in the mean & SD of speech perception test results and the aided P1 latency, amplitude of cortical evoked potential between the two groups. There was negative correlation between P1 latency and speech perception tests and a positive correlation between P1 amplitude and speech perception tests in both groups.***

**Conclusions:**

The cortical evoked potential is correlated with the speech perception ability which can help in objective prediction of speech perception abilities in CI children.

## Introduction

The important issue of the sensorineural hearing loss (SNHL) in children is its impact on the ability of speech perception (1).

Thus,an amplification device and a rehabilitation program are crucial fordeveloping speech in children with severe to profound SNHL. The most effective neural prosthesis for supplying auditory inputs to those with severe to profound SNHL is cochlear implants (CIs) (2-4).

The CI device converts acoustic energy into electrical signals that are delivered directly to the auditory nerves via biphasic pulse trains that have particular temporal and spectral characteristics (5-6).

After that the brain stem and auditory cortex detect and decode the complex speech stimulus. Children using CI exhibited significant changes in their ability to perceive speech, which is fundamental for developing speech and language (6).

The assessment of the speech perception abilities is an important step in rehabilitation and counseling of children with CI. There are various speech perception tests available; however, these tests are subjective, needs co-operation from the tested child and time-consuming. 

It may be possible to use Cortical Auditory Evoked Potentials (CAEPs) to gain insight in to

speech processing at the level of the cortex and to determine how the neural activity parallels up in the cortex (7).

Cortical processing is variable among CI users and this can lead to wide range of variation in speech perception abilities (7). The ability to perceive speech is the most important sign of normal speech and language development. Therefore, the rationale of the current study is to measure the speech perception abilities in those children subjectively and objectively.

## Materials and Methods

**I) Participants:**

Sixty children with a cochlear implant were included in this research. With the following inclusion criteria; age ranges from five to eight years, with pre-lingual bilateral profound sensorineural hearing loss, fitted with a unilateral cochlear implant MED-EL (Opus II) for two years or more, with at least eighty percent of the electrodes were active. No amplification to the other ear was available. The age of implantation was around two and half years for all children. They have average intelligence and normal middle ear condition. All children had average aided CI response via pure tone audiometer (mean 28.6 ^+^_-_ 2 SD) and aided speech perception tests (mean 26.5 ^+^_-_ 2 SD). All participated children received regular speech therapy within three months post-operative in a rate of two sessions per week each one for half an hour for at least 18 months, the method of auditory rehabilitation was the auditory training. The parents of all children were cooperative in performing listening exercises. The exclusion criteria was auditory neuropathy, nerve deficiency in Magnetic resonance imaging, other disability such as attention deficit hyperactivity disorder (ADHD) or autism.

The children were divided into two equal groups, thirty children in each; group I with good progress in auditory training and language acquisition and group II with poor progress in auditory training and language acquisition (the progress was noticed by parents in daily activity and by the speech therapist in the sessions). 

**II) Procedure:**

All the participants included were subjected to the following procedures:

1. ***Research ethics***

After parents of all children were informed about  the reasons for the study, written consent was obtained. All work was carried out in accordance with the Code of Ethics of the World Medical Association (Declaration of Helsinki) for human experiments. In addition, was approved by the Ethical Committee of the Faculty of Medicine, Sohag University, Egypt.

1. ***Clinical Trials Registry***

The research has been reviewed and approved by Thai Clinical trial Registry (TCTR) Committee on 08 April 2021. The TCTR identification number is TCTR20210408003.

1. ***Clinical examination: ***

Including otologic examination, and general examination to rule out any associated medical problems.

1. ***Aided CI response:***

Done in a sound treated room (ANSI 3X 76).

a. Aided pure tone audiometer: were measured using a calibrated Amplaid 309 clinical audiometer. Aided air conduction thresholds were measured for frequencies from 500 to 4000 Hz using loud speaker. 

b. Aided Speech audiometer: Speech reception threshold (SRT) were calculated using Arabic spondaic words for children (6).

1. ***Immitancemetry:***

Measured by AZ7 Clinical Impedance audiometer. Tympanometry was measured by the low**-** frequency tympanometry with a probe tone of 226 Hz to exclude any middle ear pathology.

1. ***Speech perception tests: ***

**A- Presentation of the stimulus:**

The test took place in a sound-proofed room with few visual and aural distractions. The test room's lighting should be adequate to allow for clear visibility of the picture plates. The test was conducted over a loud speaker with a live voice. Loudspeaker in the study group was angled 45-degree to the side of the CI. For the matched youngsters with normal hearing in the control group, the speaker was placed on the same side.

**B- Seating arrangement:**

To prevent any visual clues, the youngster and the examiner were sitting next to each other, with the examiner's chair somewhat behind the child's chair. For cochlear implanted users, the examiner sat on the implanted side. (8). 

**C-Speech material:**

**i- Presentation level:**

Speech stimuli were delivered at an usual conversational volume (approximately at 70 dB SPL).

ii- Type of speech: 

Arabic early speech perception test, which is consisted of three components.

Components of the test:

1. The First component: consists of twelve items with monosyllable, bisyllable, and trisyllable words were utilized to measure pattern perception. Each word is presented twice; if a word with the same stress pattern is chosen, it is counted right for pattern perception. 

The objective is to identify temporal patterns, therefore the word does not have to be correctly identified to be graded as correct. On the scoring sheet, words with comparable temporal categories were under-marked; a perfect score is 24 accurate words.

2. The second component tests word recognition skills and consists of twelve spondee/bi-syllabic structure and a wide range of vowels and consonants. The words were given in a random order until each word was presented twice. The youngster responds by pointing to the image that corresponds to the uttered word. A plus (+) sign was supplied if the word was correctly identified, and a negative (-) sign was given if it was not. On this test, a perfect score is 24 words correctly selected. 

3. The third component is the presence of twelve closed sets of monosyllabic words. It was created to give a more difficult condition for word recognition. This component has twelve words that are very similar. The spondee/bi-syllabic identification sub-test was used to rate responses to the monosyllable identification sub-test.

1. ***Auditory evoked potential: ***

Done by Biologic Instrument.

a- Child preparation: 

CI was ensured to be in good functioning order and to meet the required standards (9). It was also made sure that CI was adjusted to the most comfortable setting possible.

Using an abrasive gel, clean the electrode attachment sites (NeuroPrep). Silver chloride electrodes (AgCl) were placed at the recording sites after electroencephalography (EEG) paste and surgical adhesive tape were used to fix the electrodes firmly in place.

The active electrode was attached to CZ and connected to the pre-amplifier's input positive, whereas the reference electrode was attached to the test ear lobule A2 and connected to the preamplifier's input negative. The ground electrode was connected to the FPz's ground position.Children were asked to lie down quietly with minimal body movement as the adult walked around. We permitted the child to view a silent animation on the tablet screen to keep him quiet, which had no effect on the P1-N1-P2 wave-form. All participants were instructed to blink their eyes as little as possible.

b- Test parameters: 

Transducer: TDH 39 earphones, the speech processor's microphone was positioned directly on the TDH 39 earphone (10 cm).

Type of Stimulus: speech sound /ba/ 30-100 milliseconds, with rise/fall time 20 milliseconds and plateau 20 milliseconds.

Stimulus level: 70 dB nHL and the inter-stimulus interval 1125 ms. 

EEG filter: ranges from 1 Hz to 30 Hz. 

Recording time window: 100 milliseconds pre-stimulus and 600 milliseconds post-stimulus. 

Artifact rejection: from 100 to 150 mv.

Number of sweeps: 300. (10-12).

c. Interpretation:

Waveform: The waveforms’' peak potentials are labeled P1, N1, P2, and N2. Amplitude: The difference between the 0.0 UV point and the greatest positive value, which occurred between 50-300 milliseconds following the auditory stimulus to the ears (13). 

In the current study, we used normative parameters of our laboratory when we interpret the data of auditory evoked potentials; wave-forms were P1, N1, P2, and N2, latency of P1 ranges from 87.5 to 96.3 and P amplitude ranges from 1.19 to 1.27.

1. ***Statistical analysis:***

IBM SPSS Statistics for Windows version 20 was used to examine the data. Means + standard deviation are used to express quantitative data. Numbers and percentages are used to express qualitative data. The two groups were compared using correlation tests. In all statistical tests employed in the study, a 5% level of significance was chosen as the level of significance.

## Results

The sixty CI children included in this research were divided into two equal groups; Group I with good progress in auditory training and language acquisition and group II with poor progress in auditory training and language acquisition As regards the age in Group I the mean of age was 7.43 with SD 1.39 while in group II there mean of age was 7.21 with SD 1.48 (Table1,2).

**Table 1:** The mean & SD of the speech perception test results and the aided P1 latency, amplitude between the two groups

**Test**	**Group I** **No 30 Mean & SD**	**Group II** **No 30 Mean & SD**	**P**
Speech 1	17.33+- 6.92	8.17+- 4.28	< 0.0001*
Speech 2	17.93+- 2.71	8.03+- 2.59	< 0.0001*
Speech 3	8.7+- 1.596	4.30+- 1.32	< 0.0001*
P1 latency	103.50+- 55.25	147.97+- 327.77(SE: 3.31)	< 0.0001*
P1 amplitude	1.004+- 0.013	0.45+- 0.015	< 0.0001*

There was a spastically significant difference in the mean & SD of speech perception test results and the aided P1 latency, amplitude between the two groups. N.B. SE was calculated for P1 latency in Group I as the SD was higher than mean.

As regards wave-form of aided auditory evoked potential; in Group I it was normal in 25 (83.33) children and abnormal in only 5 (16.67) children. In Group II it was normal in 7 (23.33) children and abnormal in 23 (76.67) children.

**Table 2:** The correlation between speech perception score and the aided P1 latency & amplitude in Group I

**Correlation **		**P1 latency**	**P1 amplitude**
Speech 1	R	-0.1935	0.3121
	P	0.306859	0.093144
Speech 2	R	-0.2002	0.2517
	P	0.289304	0.179674
Speech 3	R	-0.7346	0.5295
	P	< .00001 *	0.002621

There was negative correlation between P1 latency and speech perception tests, only significant for category 3 of speech tests. There was positive correlation between P1 amplitude and speech perception tests.

There was a statistically significant correlation between speech tests and P1 latency & amplitude, which was negative for latency and positive for amplitude (Table 3).

**Table 3:** The correlation between speech perception score and the aided P1 latency & amplitude in Group II

		**P1 latency**	**P1 amplitude**
Speech 1	R	-0.6744	0.5593
	P	.000044	.001313
Speech 2	R	-0.6455	0.6061
	P	.000119	.000386
Speech 3	R	-0.8933	0.8921
	P	< .00001*	< .00001*

## Discussion

Children with bilaterally severe or profound hearing loss benefit from cochlear implants, which provide important auditory cues that improves their speech perception. However, many clinical studies showed that the outcome among implant recipients is highly variable. The measurement of speech perception abilities in children with CI is very crucial for audiologist. In our study, we used both subjective and objective measures to evaluate the speech perception abilities in CI children. This was done on two groups of CI users; Group I with good progress in auditory training and language acquisition and group II with poor progress in auditory training and language acquisition (the progress was noticed by parents in daily activity and by the speech therapist in the sessions). 

All children had the same inclusion criteria as regards age ranges (5: 9 years), unilateral CI MED-EL (Opus II) for two years or more, with at least eighty percent of the electrodes were active. No amplification to the other ear was available. The age of implantation was around two and half years for all children. They have average intelligence and normal middle ear condition. All children had average aided CI response via pure tone audiometer (mean 28.6^+^_-_ 2 SD) and aided speech perception tests (mean 26.5 ^+^_-_ 2 SD). 

All participated children received regular speech therapy within three months post-operative in a rate of two sessions per week each one for half an hour for at least 18 months, the method of auditory rehabilitation was the auditory training. The parents of all children were cooperative in performing listening exercises. Auditory neuropathy, nerve deficiency in Magnetic resonance imaging and other disability such as attention deficit hyperactivity disorder (ADHD) or autism were excluded.

In the present study, we compare the mean and standard deviation of both subjective and objective measures of speech perception abilities between both studying groups.

The N1-P2 complex is generally observable in adults because cortical responses change with age; however, the P1 component of cortical potential serves as a primary auditory developmental marker in children (14). P1 is responsible for recording acoustic characteristics of sound, such as frequency and time (15). As a result, the wave-form, latency, and amplitude of P1 of the cortical potential were investigated in this work.We found that there was a spastically significant difference in the mean & SD of speech perception test results and the aided P1 latency, amplitude between the two groups (Table 1).

The central auditory system changes after CI with an improvement of speech perception abilities. Speech perception abilities were related to changes at the cortical level. Meanwhile, these changes were variable among children and it was related to the subjective changes in auditory and language acquisitions. Ponton et al. who reported that in a group of implanted infants with good spoken language perception, the cortical evoked potential is clearly visible. As a result, they concluded that neuro-maturation has a major impact on the cortical potential waveform and latency value (16). In terms of the P1 waveform, we discovered that Group I exhibited a considerable robust waveform of cortical potential as well as good language acquisition and speech perception test performance. On the other hand, in Group II children with poor speech acquisition and perception scores, we discovered an altered and aberrant P1 waveform. Modifications in neural synchronization and strengthening of neural connections were linked to increased speech perception abilities, and these alterations in waveform shape were attributed to them. Purdy et al. discovered that improved behavioral speech perception scores were associated with increased cortical evoked potential resilience (10). In studying the correlation between speech perception tests and cortical evoked potential, we found that in Group I (Table 2) there was negative correlation between P1 latency and speech perception tests, only significant for category 3 of speech tests. This means that the P1 latency decrease as the speech perception test results improved. The correlation between P1 amplitude and speech perception tests was positive, which means that high speech perception test was associated with the larger amplitude. In Group II we found that there was a statistically significant correlation between speech tests and P1 latency & amplitude, which was negative for latency and positive for amplitude. This was agreed with Kelly et al. who reported that Cortical evoked potentials, which are connected to speech perception capacity and so provide objective evaluations of central auditory processing, can be elicited by complex sounds like speech (17). 

## Conclusion

In conclusion, improvement in speech perception abilities is attributed to central auditory plasticity. The subjective speech perception tests and the cortical evoked potential were correlated with the speech perception ability of CI children. Either subjective or objective assessment of speech perception abilities in those children can help in assessment of their speech progression. 

In addition, either these assessment procedures can be used as a predictive tool for evaluation of speech perception abilities in CI children. 
